# Inflammation in Post-Traumatic Stress Disorder (PTSD): A Review of Potential Correlates of PTSD with a Neurological Perspective

**DOI:** 10.3390/antiox9020107

**Published:** 2020-01-26

**Authors:** Tammy D. Kim, Suji Lee, Sujung Yoon

**Affiliations:** 1 Ewha Brain Institute, Ewha Womans University, Seoul 03770, Korea; damih.m.kim@gmail.com (T.D.K.); suji.j.lee@gmail.com (S.L.); 2 Department of Brain and Cognitive Sciences, Ewha Womans University, Seoul 03770, Korea

**Keywords:** inflammation, oxidative stress, post-traumatic stress disorder, cytokines, neuroimaging, magnetic resonance imaging

## Abstract

Post-traumatic stress disorder (PTSD) is a chronic condition characterized by symptoms of physiological and psychosocial burden. While growing research demonstrated signs of inflammation in PTSD, specific biomarkers that may be representative of PTSD such as the detailed neural correlates underlying the inflammatory responses in relation to trauma exposure are seldom discussed. Here, we review recent studies that explored alterations in key inflammatory markers in PTSD, as well as neuroimaging-based studies that further investigated signs of inflammation within the brain in PTSD, as to provide a comprehensive summary of recent literature with a neurological perspective. A search was conducted on studies published from 2009 through 2019 in PubMed and Web of Science. Fifty original articles were selected. Major findings included elevated levels of serum proinflammatory cytokines in individuals with PTSD across various trauma types, as compared with those without PTSD. Furthermore, neuroimaging-based studies demonstrated that altered inflammatory markers are associated with structural and functional alterations in brain regions that are responsible for the regulation of stress and emotion, including the amygdala, hippocampus, and frontal cortex. Future studies that utilize both central and peripheral inflammatory markers are warranted to elucidate the underlying neurological pathway of the pathophysiology of PTSD.

## 1. Introduction

Post-traumatic stress disorder (PTSD) is a chronic debilitating condition that results from having been exposed to trauma. In the current medical field, the diagnostic criteria of PTSD are largely dependent on the clinical symptomatology that outlines the disorder, including cognitive, behavioral, and affective domains [1]. Specifically, the diagnosis for PTSD includes the presence of symptoms of re-experience, avoidance, negative alterations in cognition and mood, and alterations in arousal and reactivity following the trauma [2]. Clinical symptoms of PTSD have been the sole standard for its diagnosis as acquired through self-report and/or structured clinical interview, and have been demonstrated as reliable predictors of a number of important health outcomes. For instance, previous studies have shown that PTSD symptoms predict the degree of functional recovery from pain [3], depressive symptoms [4], risk of substance abuse [5], and factors related to quality of life such as feeling of dissatisfaction [6]. Furthermore, in addition to the psychobehavioral clinical features, PTSD often involves comorbidities with other health problems such as obesity [7], type 2 diabetes mellitus [8], and cardiovascular complications [9]. Considering that all of the above health concerns involve problems in metabolic syndrome which are then closely related to oxidative stress and inflammation, it may then be presumed that the underlying mechanisms of PTSD involves the dysregulation of the immune system.

While psychobehavioral symptoms are the major factors considered when investigating the pathological state and severity of one’s PTSD, understanding the immunological alterations that occur in tandem with these symptoms may be informative. For instance, detailed inflammatory responses to various oxidative stress in PTSD such as physical trauma and psychosocial stress may help identify the potentially distinct or diverging pathways towards the development of PTSD, and further reveal the pathophysiology of the disorder [10]. Growing research on PTSD recommends the development and promotion of early treatment strategies for PTSD prior to any clinical symptom development [11], as trauma-exposed individuals with high PTSD symptom severity demonstrated to have less likelihood of seeking treatment [12], resulting in a higher risk of chronic PTSD. For studies that investigated the biological factors associated with the clinical symptoms of affective or anxiety disorders, findings have shown that clinical symptoms in patients with depression [13] and anxiety disorders [14] are significantly associated with altered profiles of oxidative stress and inflammation, such as altered serum inflammatory markers. Interestingly, these alterations in inflammatory markers remained significant as compared to individuals in their respective control group, even after adjusting for demographic variables and lifestyle, suggesting that inflammatory response within the central nervous system (CNS) may be partly involved in the pathophysiology of PTSD. Furthermore, some studies have indicated that signs of inflammation may not necessarily correlate with the clinical symptoms of PTSD in a direct manner [15,16], and therefore the inclusion of one’s immunological state along with the clinical symptom and severity of PTSD may provide further details of one’s diagnostic state for PTSD in a more comprehensive manner. Moreover, considering that PTSD also has a late-onset tendency for clinical symptom development [17], incorporating data on the inflammatory state in the acute aftermath of trauma may help promote various strategies of early prevention and intervention for PTSD, as opposed to being solely dependent on the emergence of clinical symptoms as markers of the disorder.

In recent years, a growing number of studies have investigated the immunological alterations in PTSD as to elucidate the detailed biological pathways that underlie the clinical symptoms of the disorder. In particular, in addition to the abundant number of animal model and in vitro studies on the matter, studies using a human cohort have emerged based on the latest version of the Clinician-Administered PTSD Scale as according to the fifth version of the Diagnostic and Statistical Manual (CAPS-5) [18]. Techniques of neurological assessments including magnetic resonance imaging (MRI) and positron emission tomography (PET) have also emerged, which may allow to investigate the inflammatory responses in PTSD from a deeper, neurological perspective. For instance, previous systematic reviews have been done on MRI-based studies that demonstrated significant structural [19] and neurochemical [20] alterations in specific regions of the brain in association with stress or trauma. Further research that investigate these neurological alterations in PTSD in association with inflammatory responses as well as specific clinical symptoms of PTSD using up-to-date methods of clinical assessment may further reveal the underlying pathways of the disorder in a multi-level perspective.

A number of reviews have also been published that discuss the inflammatory changes in a human PTSD model. In particular, articles investigated inflammation as observable through specific serum inflammatory markers that are known to be in association with PTSD [21,22] and conducted meta-analysis and meta-regression [23] as to summarize elevated systemic levels of oxidative stress and inflammation in individuals with PTSD as compared to healthy controls. However, reviews on the topic of inflammation and PTSD have demonstrated inconsistent findings, which have been suggested to be the result of the heterogeneity of the disorder. While controlling for all the factors that make up the heterogeneity of PTSD across the studies reviewed may be difficult to conduct, a targeted and structured review that excludes some of the major contributors to the heterogeneity of PTSD may help reveal some of the detailed underlying pathways of PTSD in relation to inflammation, such as the exclusion of PTSD with comorbid complications, and observing for a potential pattern according to various types of trauma. Moreover, research also indicates that the time between the onset of trauma and the time of assessment may be an important factor that influences the heterogeneity found in inflammatory response in PTSD [24], therefore the distinction between early-onset and later onset of trauma exposure should be considered in addition to recent reviews. A comprehensive and updated review on the growing literature that were recently published on inflammation in PTSD according to various factors that potentially contribute to the heterogeneity may explain for the contrasting findings that were discussed to date. Considering that a few studies in the past have suggested that specific inflammatory markers may play an important role specifically in PTSD [25] and previous articles suggested specific biomarkers as promising for indicating inflammation in PTSD [21], summarizing findings from recent literature on such specified markers may help identify potential biological markers that indicate one’s pathophysiology of PTSD.

The current study aims to provide a review of the recent literature on the inflammatory aspect of PTSD in a human model and discuss potential ways to better understand one’s state of PTSD through the additional perspective of oxidative stress and inflammation. Specifically, we will review the physiological changes that occur in PTSD as measurable through inflammatory biomarkers, as well as neurological aspects of inflammation in PTSD through neuroimaging methodologies. The summary of the major findings of PTSD with respect to oxidative stress and inflammation may promote further discussion on the potential ways of early detection of those who are at risk for PTSD, as well as early intervention strategies for PTSD.

## 2. Materials and Methods

### 2.1. Literature Search Strategy

A literature search was conducted using two major electronic databases: PubMed (https://www.ncbi.nlm.nih.gov/pubmed/) and Web of Science (https://clarivate.com/webofsciencegroup/solutions/web-of-science/). The search was performed under a filter such that studies published from January of 2009 through November of 2019 were included. The following keywords were used as search terms: (1) “inflammation” AND “PTSD”, (2) “inflammation” AND “PTSD” AND “magnetic resonance”, (3) “oxidative stress” AND “PTSD”, (4) “oxidative stress” AND “PTSD” AND “magnetic resonance”. Records obtained from the literature search were reviewed such that duplicates of articles identified between the two databases were removed, and the remaining records were assessed for eligibility and excluded accordingly, under the following eligibility criteria.

### 2.2. Eligibility Criteria

Studies included in the current review were first screened such that they were original research articles using a human cohort, with a target population of PTSD that also explored the alteration in inflammatory state. The following records were to be excluded as part of the screening process: (1) records that are not original research including review papers, conference abstracts, short communications, or commentaries, (2) studies that used animal models, in vitro models, or post-mortem studies, (3) records that were not in English language, or (4) study designs that did not investigate inflammation in a target population of PTSD. After the screening process, the eligibility of the remaining articles was assessed using a full-text review. Articles that met the following criteria were further excluded: (1) studies that investigated PTSD with other comorbid disorders, diseases, or medical conditions (i.e., bipolar disorder, major depressive disorder, cardiovascular disease, myocardial infarction, etc.), or (2) studies that performed clinical trials or other forms of intervention as part of the study design. In addition, studies that investigated the inflammatory state in PTSD from a pediatric sample population were excluded, as well as target population of childhood onset trauma, as previous literature noted that the time lag between the time of trauma and time of assessment or sampling may influence the degree of alteration in inflammatory cytokine levels [24].

### 2.3. Inflammatory Markers of Interest

For the current review, a select number of inflammatory markers that were previously described as potential biomarkers of PTSD [26,27] were targeted as methods of assessment for inflammation. Studies that investigated the alterations in these selected inflammatory markers were considered eligible, including levels of proinflammatory and anti-inflammatory cytokines. Specifically, cytokines that are most prevalently observed as being altered in relation to PTSD were selected as follows: serum proinflammatory cytokines interleukin (IL)-1β, IL-6, tumor necrosis factor (TNF)-α, and interferon (IFN)-γ as well as C-reactive protein (CRP) which is influenced by proinflammatory cytokines [28], serum anti-inflammatory cytokines IL-4 and IL-10 [29], and PTSD- and oxidative stress-related genes including the brain-derived neurotrophic factor (*BDNF*), arachidonate 12-lipoxygenase (*ALOX12*), arachidonate 15-lipoxygenase (*ALOX15*), and retinoic acid orphan receptor alpha (*RORA*) [30]. Neuroimaging-based studies were also included such as MRI and PET, along with markers of inflammation to examine the moderating role of specific inflammatory markers in the alteration of the brain.

## 3. Results

### 3.1. Results from the Literature Search

**Figure 1:**
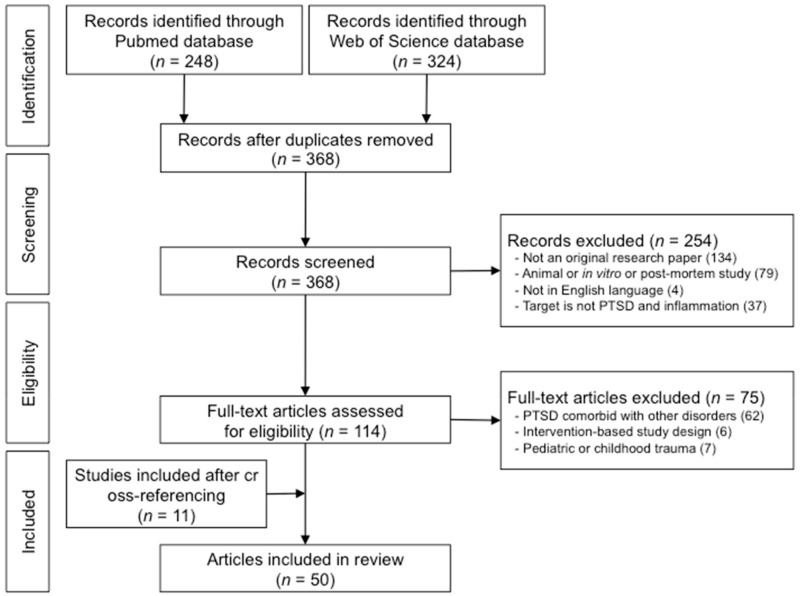
Flowchart of literature search and identification of eligible articles for the current review.

A total of 572 articles were identified after the literature search conducted under the databases PubMed and Web of Science. Upon the search and identification of records, duplicate records between the two databases were removed, followed by the screening of titles and abstracts of the studies. The screening process excluded a total of 254 studies as according to the eligibility criteria mentioned above in the Materials and Methods section. After careful consideration and full-text review of the remaining records according to the eligibility criteria, 75 studies were removed. After the inclusion of 11 studies through cross-referencing and reviewing of the records, and a total of 50 studies were included in this review. Individuals with PTSD who were participants of studies that were included in this review were exposed to various types of trauma, including accidents such as surviving a car crash, fire, or natural disaster, victims of various crimes including physical assault, sexual violence, or domestic abuse, exposure to combat such as military personnel or police officers, and other forms of trauma that were not specified. Among the 50 articles selected for this review, 43 were studies that explored serum inflammatory cytokine markers as measures of inflammation, 4 were studies exploring PTSD- and oxidative stress-related genes, while the remaining 3 were neuroimaging-based studies that explored the neural correlates of inflammation in PTSD. A detailed summary of the literature search process and the screening and inclusion of the eligible studies for the current review is shown in Figure 1.

### 3.2. Alterations of Serum Inflammatory Markers in Association with PTSD

Growing literature emphasizes the importance of regulation of mental health and stress in various disorders including PTSD due to the high prevalence of comorbidity with other chronic metabolic diseases [31]. For instance, the presence of PTSD has been noted to co-occur or associate with risks of chronic medical diseases including cardiovascular diseases and type 2 diabetes mellitus [31]. Considering the significance of cytokines as regulators of immune responses [32], the majority of studies explore the alteration and influence of inflammation in PTSD through the measurement of peripheral cytokine levels. In particular, serum cytokines are often measured and used as peripheral markers of inflammation in various psychiatric disorders including depression [33], panic disorder [34], bipolar disorder [35], and obsessive-compulsive disorder [36].

Cytokines are largely divided into two classes, which are the proinflammatory cytokines and the anti-inflammatory cytokines. As demonstrated via animal models, proinflammatory cytokine levels tend to reflect the promotion and occurrence of inflammation whereas anti-inflammatory cytokine levels are related to the suppressing of inflammation as well as the protection of further damage from inflammation [37]. For studies investigating inflammation in PTSD according to peripheral inflammatory markers, the current review identified and selected 24 studies that explored serum levels of proinflammatory cytokines including one or more of IL-1β, IL-6, TNF-α, and IFN-γ as well as CRP, and 19 studies that explored serum levels of anti-inflammatory cytokines IL-4 and/or IL-10.

#### 3.2.1. Roles of Proinflammatory Cytokines in PTSD: IL-1β, IL-6, TNF-α, IFN-γ and CRP

Proinflammatory cytokines tend to be the more prevalently used outcome variables, as increased proinflammatory cytokines have consistently demonstrated to indicate worsening of the immune system, and may therefore be reliable and direct indicators of disease progression [32]. In addition, despite serum proinflammatory cytokines being peripheral in nature, they have been described as mediators of many pathways underlying the CNS through its important roles in inflammatory response [38], therefore may provide valuable insight with regards to the pathophysiology of PTSD from a CNS perspective. Proinflammatory cytokines increase in concentration with age but also may be elevated with stress as well as secondary symptoms of stress such as sleep and fatigue [24]. The current study summarizes the collective findings from three of the most prevalently explored proinflammatory cytokines in PTSD, which are IL-1β, IL-6, and TNF-α, all of which are often paired as they are all induced by the same endotoxin, lipopolysaccharides (LPS) [39], along with other inflammatory measures. Furthermore, among the 4 proinflammatory cytokines and CRP selected as part of this review, IL-6 downregulates the synthesis of IL-1β and TNF-α [40]. Considering that a distinct regulatory relationship is present among the inflammatory markers of interest of the current review, it may be presumed that alteration in the markers provides implications with regards to the potential direction of alteration in the other remaining cytokines as well.

Through the current literature selection, studies have demonstrated that individuals with PTSD have significantly elevated serum levels of proinflammatory cytokines than the respective control group without PTSD. Specifically, individuals with PTSD demonstrated as having higher levels of serum IL-1β [15,25], IL-6 [15,16,25,41,42,43,44,45,46,47], TNF-α [15,16,25,42,43,44,46,47,48], IFN-γ [15,47,49], and CRP [42,50] than individuals who have been exposed to trauma but have never developed PTSD. In the current review, IL-6 demonstrated to be the most documented proinflammatory cytokine in human models of PTSD, and the majority of studies demonstrated increased IL-6 levels in the serum sample of individuals diagnosed with PTSD as compared with their respective control group. This may also be in alignment with previous research which noted IL-6 as the most considerable marker for inflammation [21]. Specifically, studies have shown that IL-6 is crucial in the relationship between immune system and CNS in inflammatory states [44]. Furthermore, 4 studies have demonstrated that individuals with PTSD have elevated proinflammatory cytokine levels when compared with healthy individuals who have not been exposed to any trauma [51,52,53,54]. In addition, studies have also demonstrated elevated CRP levels which are known to be influenced by proinflammatory cytokines, where individuals with PTSD showed increased CRP levels compared to healthy controls [42], and PTSD symptom severity were positively associated with CRP levels [50,55].

Participants with PTSD resulting from surviving a fire and burn injury were assessed for proinflammatory cytokines including IL-1β, IL-6, IL-8, and TNF-α in a study by Jiang and colleagues [51]. Here, Jiang and colleagues (2018) have also noted that, individuals with severe burn injury and diagnosed with PTSD demonstrated decreased clinical symptoms of PTSD three months after the trauma exposure, while having remained elevated levels of proinflammatory cytokines [51]. In addition, a study by Gill and colleagues (2013) found that women who recover from PTSD have statistically similar IL-6 levels as healthy control individuals who have never been exposed to any trauma [52]. This may then indicate that, for individuals who are able to recover from PTSD, both the normalization of clinical symptoms of PTSD as well as the influences of oxidative stress and inflammation may be possible. Furthermore, the findings from Jiang and colleagues (2018) on the sustained elevation of proinflammatory cytokine levels despite having reduced in clinical symptoms of PTSD [51] may indicate that those who are susceptible or possibly at risk of chronic PTSD may have distinct inflammatory responsiveness, regardless of clinical symptomatology. Therefore, the distinct pattern between inflammatory measures and clinical measures may warrant the incorporation of both physiological data as well as clinical assessments to better understand the pathophysiology of PTSD.

The study by Wang and colleagues (2016) may provide supportive evidence to the potential distinct inflammatory pathways between individuals who are at risk for versus resilient to PTSD [46]. In particular, their findings indicated that the severity of the trauma experienced may not necessarily mediate or influence the relationship between level of serum proinflammatory cytokine IL-1β and post-traumatic stress symptom severity in the case of combat-related PTSD [46]. Among veterans all of whom have been exposed to similar combat, those who are diagnosed with PTSD demonstrated significant imbalance in their inflammatory state as compared to those without PTSD [46], implicating that one’s risk or resilience towards PTSD may be largely explained by biological factors and their ability to regulate their inflammatory state, as opposed to the nature or severity of the traumatic event.

Another interesting finding from the studies identified is the supportive evidence of stress influencing proinflammatory cytokine levels even at a younger age group, as well as the potential predictive role of proinflammatory cytokine levels in the comorbidity of PTSD and other chronic metabolic diseases. In particular, Blessing and colleagues (2017) demonstrated increased indicators of systemic inflammation in individuals with PTSD as compared to the respective control group, where the inflammatory markers were also indicators of higher cardiovascular risk as well as insulin resistance [43], which may then provide additional perspective with regards to related diseases such as type 2 diabetes mellitus.

Furthermore, two studies also explored the additional factor of traumatic brain injury (TBI) in PTSD and found significant alteration in proinflammatory cytokine levels in association with PTSD. The first is the study by Devoto and colleagues (2017) who found that TBI may have an additive effect to the alteration of proinflammatory cytokine levels in the case of PTSD, as individuals with PTSD as well as TBI had demonstrated greater elevation in proinflammatory cytokine levels than individuals with PTSD but without TBI [44]. The study also demonstrated that, among individuals with both PTSD and TBI, the levels of proinflammatory cytokines were correlated with post-traumatic stress symptom severity, where serum proinflammatory cytokine levels of IL-6 and TNF-α were higher in individuals with higher post-traumatic symptom severity. The second study is by Kanefsky and colleagues (2019), which further demonstrated that, even within individuals with both PTSD and TBI, the presence of loss of consciousness at the time of the traumatic brain injury may also influence the alteration in proinflammatory cytokine levels of IL-6 [45].

Contrasting findings were also reported in this literature search. For instance, studies have reported that victims of assault who are exposed to trauma and have been diagnosed with PTSD have statistically similar IL-6 cytokine levels as those who have been exposed to trauma but have not developed PTSD [56]. In addition, one study reported insignificant alteration in three of the serum proinflammatory cytokine levels between individuals with PTSD versus those without PTSD [57], and another study reported insignificant alteration in IL-6 in relation to PTSD [58]. Moreover, a study reported small but significant decrease in the levels of IL-6 and IFN-γ in individuals with PTSD as compared to those without PTSD [59]. However, it may be noteworthy that these studies were primarily focused on stress as measured by symptoms of insomnia, respectively, rather than the alteration in the general symptoms PTSD through oxidative stress as measured by the proinflammatory cytokines targeted in this review.

Similarly, McCanlies and colleagues (2011) reported that there was a lack of association between proinflammatory markers of IL-6 and CRP with onset of PTSD symptomatology in urban police officers with high PTSD symptoms [60]. There were also opposite findings reported in one study by Bruenig and colleagues (2018), which reported a positive correlation between post-traumatic stress symptom severity and reduced levels of serum TNF-α [61]. Despite the numerous reports of contrasting findings in terms of levels of proinflammatory cytokines according to the diagnosis of PTSD, however, it is noteworthy that the majority of the literature selected for review upon the screening and assessing for eligibility were largely based on male populations. Considering that between-sex differences in the levels of proinflammatory cytokines IL-6 have been reported [54], where men with PTSD show significantly elevated IL-6 levels as compared to their respective healthy control group while the women did not, this may be an important factor to consider in the summary of the studies selected in this review.

A detailed description of the studies reviewed that investigated alteration in serum proinflammatory cytokine levels in relation to PTSD is provided in Table 1.

#### 3.2.2. Roles of Anti-Inflammatory Cytokines in PTSD: IL-4 and IL-10

In addition to proinflammatory cytokines, anti-inflammatory cytokines also play a pivotal role in the regulatory processes of oxidative stress and inflammation. In contrast to proinflammatory cytokines, which promote and induce inflammatory responses, the primary role of anti-inflammatory cytokines is to inhibit the synthesis of proinflammatory cytokines, and is therefore also described as inhibitors of inflammatory mediators [32].

While numerous anti-inflammatory cytokines are present, the current study reviewed the alteration in levels of IL-4 and IL-10, as it has been previously suggested as an important marker in psychosocial stress [27] as well as other chronic medical diseases including type 2 diabetes mellitus [62] and cardiovascular disease [63], both of which are more often than not closely associated with PTSD. For instance, previous studies have consistently reported that level of serum anti-inflammatory cytokine IL-10 is significantly associated with insulin sensitivity in young individuals, which may later develop towards type 2 diabetes mellitus [64] or chronic pain [65]. In addition, a recent study has also reported that chronic inflammation as observable by altered levels of anti-inflammatory cytokine levels including IL-4 and IL-10 may play a predictive role in disorders such as type 2 diabetes mellitus development [66].

Furthermore, IL-10 enables the suppression of synthesis of TNF-α as well as IL-1β [34], which are proinflammatory cytokines primarily explored in models of PTSD, and may therefore provide a comprehensive overview of the specific inflammatory biomarkers that may represent the pathophysiology of PTSD. Considering that anti-inflammatory cytokines have also been suggested to be potential options of possible treatment of chronic disorders [67], an overview of the alteration in IL-4 and IL-10 may reveal important findings with regards to the distinct role of anti-inflammatory cytokines in PTSD.

A total of 19 studies were selected from the literature search process that explored the alteration in serum levels of IL-4 or IL-10 in PTSD. Contrary to expectations of the opposition roles between proinflammatory and anti-inflammatory cytokine alterations in response to oxidative stress and inflammation, findings from the selected studies have mostly demonstrated statistically similar levels of serum IL-10 between individuals with PTSD and trauma-exposed controls who have never been diagnosed with PTSD [15,16,25,44,45,57,68,69,70]. Among these studies that demonstrated no alteration in serum IL-10 also included those from Table 1 which demonstrated significant increased proinflammatory cytokine levels in the PTSD group as compared to their respective healthy controls [15,16,25,44,45], indicating that elevated proinflammatory cytokines in association with PTSD may not necessarily reflect reduced anti-inflammatory cytokines. Furthermore, for studies that included a healthy control group who have not been exposed to any type of trauma, consistent results were shown where individuals exposed to trauma – regardless of their diagnosis for PTSD—did not show any between-group differences in IL-10 levels as compared to the control [69,71].

However, it is noteworthy that contrasting findings were reported by de Oliveira and colleagues (2018) as well as Guo and colleagues (2012), where both proinflammatory cytokines including IL-6 and anti-inflammatory cytokines including IL-4 and IL-10 were positively correlated and elevated in individuals with PTSD as compared to the respective healthy control group [54,72]. Dennis and colleagues (2018) also found that PTSD symptom severity is associated with higher IL-10 levels, which are then mediated by vagal activity, smoking, and alcohol dependence [48]. However, this may be partially explained by the well-distributed sex ratio in this particular study of 87 males and 80 female, considering that previous studies have noted significant sex differences in the patterns of inflammatory marker levels. Elevated IL-10 levels in respect to the diagnosis of PTSD were also found in two studies [52,54], although these studies were heavily male-dominant and female-dominant in their populations, respectively. Considering that many studies demonstrated findings of reduced IL-10 levels in association with PTSD diagnosis [46,56,61], this may then suggest that perhaps this anti-inflammatory cytokine are not a suitable marker that provides insight towards the pathophysiology of PTSD or presence of trauma exposure.

A detailed description of the studies reviewed that investigated alteration in serum anti-inflammatory cytokine levels of IL-4 and IL-10 in relation to PTSD is provided in Table 2.

### 3.3. Roles of PTSD- and Oxidative Stress-Related Genetic Markers in PTSD

Five studies explored specific genes that have been previously suggested to be of significance in PTSD and oxidative stress, which included the investigation of the *ALOX12* and *ALOX15* [74], *BDNF* [75,76], and *RORA* [77,78]. In summary, studies based on individuals who were exposed to combat-related trauma demonstrated inconsistent findings, where one reported that veterans with PTSD have higher frequencies of the Met/Met and 66Met alleles as compared with veterans without PTSD [75], while another demonstrated that the frequencies of the two alelles were similar between the veteran groups [76]. While the findings from the two studies are contrasting, it is noteworthy that both studies strictly included trauma-exposed veterans without the inclusion of a healthy control group that has not been exposed to trauma, and the studies vary in sex distribution. Furthermore, Bruenig and colleagues (2016) have noted that the low Met/Met genotype frequencies in their sample may have influenced the results also [76].

Two other studies have explored the association between single nucleotide polymorphism (SNP) in the *RORA* gene with PTSD, where both studies found the same SNP within the *RORA* gene (rs8042149) to be significantly associated with the presence of PTSD [77,78]. Considering that the *RORA* gene is expressed in neurons and brain structures as to serve neuroprotective roles within the brain such as in response to oxidative stress [77], these findings indicate that *RORA* may be a marker for assessing one’s capability in neuroprotection towards oxidative stress or inflammation, therefore a potential marker for an individual’s risk or resilience towards developing PTSD. The two genes *ALOX12* and *ALOX15* were also assessed in one study [74], which demonstrated that the *ALOX12* locus (rs1042357 and rs10852889) significantly moderated the association between PTSD diagnosis and reduced cortical thickness in the brain, whereas the *ALOX15* locus did not moderate in this association.

A detailed description of the studies exploring PTSD- and oxidative stress-related genetic markers in relation to PTSD is provided in Table 3 and Table 4.

### 3.4. Brain Alterations in Relation to Inflammation and Oxidative Stress in PTSD

Another growing field of interest within the study of inflammation and oxidative stress in PTSD is the neuroinflammatory responses within the brain. Neuroinflammation refers to the physiological reactivity of the brain in response to various types of inflammation or oxidative stress, including but not limited to external injury or trauma [79,80], and is often observed in individuals with chronic diseases during their early stages of the disease as part of a coping mechanism [81]. Considering that investigating neuroinflammatory responses are often invasive in nature [79], the utilization of neuroimaging techniques to target inflammatory responses within the brain may potentially bridge the association between altered levels of peripheral biomarkers such as proinflammatory and anti-inflammatory cytokines and the clinical symptoms that arise in PTSD.

In the case of proinflammatory cytokine, previous literature have noted that IL-1β and IL-6 are secreted at stress-induced states and take part in the catecholaminergic pathways [82]. Considering that the catecholaminergic pathway has been demonstrated to play an important role in specific regions of the brain including the hippocampus and amygdala [82], it has then been suggested that these peripheral inflammatory markers may represent alteration in the function or activity of the hippocampus and amygdala, influencing symptoms including but not limited to emotion lability [83], fear reactivity, and retrieval of traumatic memories [82], all of which are crucial factors in PTSD. Previous studies have also shown that IL-1β is known to be consistently increased in major depression after psychosocial stress [84].

Over the years, emergence of novel technologies in neuroimaging have allowed the indirect correlative investigation of neuroinflammation without the invasive nature of directly measuring neuroinflammatory responses, as neuroimaging methods allow the direct measurement of the structure and functional state of the brain. In particular, neuroimaging techniques including MRI and PET have been largely utilized as they can obtain detailed information with regards to the structure of the brain such as cortical thickness, subcortical volume, or the metabolism of targeted brain regions as measured by glucose uptake. The current search included 3 neuroimaging-based studies on oxidative stress and inflammation in PTSD. The selected studies all reported findings that were consistent with previous literature, where markers of oxidative stress or inflammation are significantly associated with alterations of the brain.

The first study is by Miller and colleagues (2015), which explored the influence of the gene *ALOX12* on the association between PTSD and cortical thickness of the brain [74]. This study takes on an approach that is distinct from peripheral measures of proinflammatory cytokines, in that it investigated the *ALOX12* locus and its potential moderating roles, based on previous knowledge that the *ALOX12* enzyme plays a key role in an oxidative-related neuronal death program [85,86]. Findings from this study first indicated that the *ALOX12* locus—through two single nucleotide polymorphisms rs1042357 and rs10852889—plays a significant moderating role in the association between PTSD and reduced cortical thickness of the brain. Specifically, brain areas with reduced cortical thickness included the middle frontal gyrus, superior frontal gyrus, rostral anterior cortex and medial orbitofrontal cortex, all of which were close in proximity and part of the frontal cortex of the brain.

The second study is by O’Donovan and colleagues (2015), which provided findings that are consistent to previous literature, where elevated proinflammatory cytokine levels of soluble receptor II for tumor necrosis factor (sTNF-RII) were significantly associated with PTSD [87]. The study also explored the influence of inflammation from a neurological perspective, and found that elevated levels of sTNF-RII is associated with reduced hippocampal subcortical volume in the case of veterans who have been exposed to combat and are diagnosed with PTSD, while this association was not present in veterans without PTSD [87]. Here, it is noteworthy that while the study also explored the potential roles of IL-6 by observing for alterations in serum IL-6 level, findings reported that IL-6 was not statistically associated with the reduced hippocampal volume in PTSD. In fact, findings indicated that higher post-traumatic stress symptom severity is associated with increased sTNF-RII and reduced IL-6 levels, which may contrast to previous literature as shown in Table 1, where the majority of individuals with PTSD were characterized with elevated serum levels of IL-6. This contrasting finding may be explained by the fact that, while IL-6 is often described as a proinflammatory cytokine marker of inflammation, it also does have anti-inflammatory properties [88,89]. Specifically, the primary roles of IL-6 are largely coupled with the regulation of the other two proinflammatory cytokines IL-1β and TNF-α, and studies have reported that IL-6 often inhibits the expression of IL-1β and TNF-α while promoting IL-10 [90]. This has been suggested to be especially so when in an anti-inflammatory environment, where the integrity of the blood-brain barrier needs to be regulated [91], such as during the occurrence of neuroinflammatory responses.

The last of the neuroimaging-based studies reviewed is a pilot study using 18F-fluorodeoxyglucose (FDG)-PET by Toczek and colleagues (2019), which investigated the association between the levels of proinflammatory cytokines and the amygdala of the brain as to explore the relationship between oxidative stress and inflammation measurable by peripheral markers and the alteration of the amygdala [92]. The study reported that there were no significant associations between levels of IL-1β, IL-6, and TNF-α with FDG signal of the amygdala. The FDG signal has been used as a tool to detect vascular inflammation in specific regions [93] and FDG signal within a brain region may provide important contextual information such as the metabolism and glucose intake within the region [94,95], ultimately enabling the inference regarding the functional activity of the respective brain region. Although there had been no associations found between levels of IL-1β, IL-6, and TNF-α and amygdala activity as measured by FDG signal, findings from the study by Toczek and colleagues (2019) reported a significant correlation among FDG signal in the amygdala, spleen and bone marrow [92]. The spleen and bone marrow are closely related organs that are also described as lymphoid organs [96,97] and have been previously described as key factors in the immune system as immune cells migrate across the spleen [97], while the bone marrow is the production site for lymphocytes [98]. As such, while proinflammatory cytokine levels did not alter in association with FDG signal of the amygdala in this study, correlation between FDG signals within lymphoid organs and the amygdala in individuals with PTSD may implicate inflammatory responses within the brain that results from trauma. Considering that FDG signal within the spleen and bone marrow have been suggested as potential approaches to measuring systemic inflammation in other chronic metabolic diseases [99], this finding may reflect a link between amygdala activity and systemic inflammation in PTSD. The findings by Toczek et al. (2019) are also consistent with a portion of previous literature, which provided evidence for brain alterations in specific regions including the amygdala, hippocampus, and prefrontal cortex in patients diagnosed with PTSD [100,101]. In particular, previous literature suggested that the amygdala, hippocampus, and prefrontal cortex each has a high density of glucocorticoid receptors, which are then related with the activation of the hypothalamic-pituitary-adrenal (HPA) axis [102]. Considering that the HPA axis is a significant contributor in responsiveness towards psychosocial stress [103] and various forms of trauma exposure [104], studies that bridge the relationship among alteration in inflammatory markers from a genetic or peripheral level, structural and functional alterations of brain regions associated with PTSD, and the clinical symptoms of PTSD, may provide a more comprehensive overview of the pathophysiology of PTSD as a non-communicable disease.

A detailed description of the neuroimaging-based studies reviewed that investigated the relationship between inflammatory and oxidative stress markers and alteration within the brain in PTSD is provided in Table 4.

## 4. Conclusions

The current structured review provides an overview of the recent evidence presented on the influence of oxidative stress and inflammation in PTSD. The studies reviewed here demonstrate the alterations of specific peripheral inflammatory markers that may potentially be implemented as correlates of PTSD, including the elevated levels of serum proinflammatory cytokines IL-1β, IL-6, and TNF-α. Among these, IL-6 has been shown to be elevated or reduced in the serum of individuals exposed to trauma according to the source of oxidative stress, such as whether the trauma is physical in nature, includes the presence of TBI or loss of consciousness, or entails psychosocial trauma. This may in turn suggest the potential of serum IL-6 level as a peripheral marker for PTSD based on trauma type. This review also discussed the three neuroimaging-based studies on the inflammatory response associated with PTSD within the brain, from which can be concluded that particular brain regions may be identifiable as neural correlates of PTSD, including the prefrontal cortex, hippocampus, and amygdala of the brain. Specifically, the evidence from the studies reviewed revealed reduction in cortical thickness among regions of the prefrontal cortex and hippocampal volume, as well as a potential link between amygdala activity and systemic inflammation in PTSD.

Given the current findings summarized, a potential pathway underlying inflammation in PTSD may be suggested. A major collective finding from this review was elevated serum IL-6 levels in all but one study [48] among the studies reviewed for alteration in proinflammatory cytokines in association with PTSD across various trauma types. Considering that IL-6 has been known to cross the blood-brain barrier (BBB) as an immune mediator [105], it may then be that psychosocial stress as a result of trauma induces elevated peripheral IL-6 concentrations, which then influences the inflammatory cytokines within the brain via crossing of the BBB. This potential pathway may also be in alignment with previous animal model study findings which reported increased proinflammatory cytokines within specific regions of the brain associated with PTSD including the hippocampus, amygdala, and prefrontal cortex [106], all of which are in alignment with the three neuroimaging-based studies reviewed in the current article also (Table 4). Moreover, the same study also reported reduced anti-inflammatory cytokines within the three brain regions [106], suggesting a similar pathway in the case of anti-inflammatory cytokines with respect to psychosocial stress in PTSD as well. The over-expression of IL-6 and other proinflammatory cytokines within the brain as a result of peripheral cytokines crossing the BBB in response to trauma and psychosocial stress may then lead to neurodegeneration as previously described [107], and thus leading to neural tissue loss followed by dysfunction in the respective brain regions. Dysfunction in the regions of the prefrontal cortex, amygdala, and hippocampus are then responsible for functions of executive control, emotional lability, fear reactivity, and retrieval of traumatic memories as demonstrated in PTSD. The dysfunction of the three brain regions according to inflammation may further be explored through detailed clinical assessments according to function, such as neurocognitive tests and PTSD symptom severity as measurable by CAPS-5. Here, the alteration in glucocorticoid receptor regulation in the three brain regions may also influence the neurochemical profile of the respective brain regions. For instance, alteration in proinflammatory cytokines including IL-6 as a result of psychosocial stress may disrupt the relationship between proinflammatory cytokines and glucocorticoid receptors, whose function is to inhibit and regulate proinflammatory cytokines [108]. Considering that disruption in glucocorticoid receptor signaling affects immune function as well as hypothalamus-pituitary axis including levels of cortisol [109], these alterations may further reinforce the related clinical symptoms of PTSD such as sustained fear and anxiety.

It is also important to note that, among trauma-exposed individuals, many individuals were found resilient and did not develop PTSD. The distinct pathways between trauma-exposed individuals who are diagnosed with PTSD versus trauma-exposed individuals who are resilient to PTSD may be broken down to two possibilities, one of which is having a preexisting proinflammatory state that distinguishes between individuals who are at risk or resilient to PTSD as described in previous studies [110,111]. Here, the premise is that individuals who are at risk for PTSD have a distinct proinflammatory state prior to any exposure to trauma as compared with resilient individuals. Another perspective is the distinct responsiveness towards trauma that may vary according to genetic factors [21], such as the *ALOX12* locus as described in Table 4 [74]. Since the activation of many genes including *ALOX12*, *ALOX15*, and *RORA* have shown to alter according to oxidative stress in a PTSD model [108], a targeted approach that observes for the relationship between inflammation and trauma exposure according to genetic variants may identify the risk or resilience factors of PTSD.

A few limitations should be noted. First of all, the current review provided an overview of some of the major peripheral inflammatory markers that are targeted in the field of PTSD research. As such, a large portion of inflammatory cytokines including chemokines have been excluded in this review in order to take a targeted, narrow approach to identifying the potential biomarkers of PTSD. In addition, considering previous literature which emphasized the significance of particular genes in relation to PTSD and inflammation beyond the currently reviewed genes, future reviews that include a wider scope of methodological approaches in this topic are warranted for a more supportive mechanistic interpretation of the findings. Furthermore, despite previous studies which suggested that immune mediators such as the proinflammatory cytokines discussed in this review are able to directly influence the CNS including the hippocampus of the brain through the crossing of the BBB [105], the current structured review included a small number of neuroimaging-based studies as supportive evidence. Future directions in the study of inflammatory responses within the brain in PTSD as well as other non-communicable diseases may benefit in further exploring the detailed alteration of the brain in response to stress through the review of various neuroimaging techniques, such as magnetic resonance spectroscopy (MRS), diffusion tensor imaging (DTI), functional MRI (fMRI), and perfusion MRI. It is also noteworthy that current literature in this topic of interest largely consist of male participants, due to the nature of conditions that are most prevalent in the exposure of traumatic events. In order to better distinguish the sex differences that had been discussed in some of the studies reviewed, future research that consider a wider distribution of target populations according to trauma type is warranted. Lastly, the literature selection criteria of the current review excluded a number of studies that provided significant evidence with regards to the pathophysiology of PTSD due to its comorbid sample population. As PTSD has a prevalence of comorbidity with a vast number of other chronic diseases [7,8,9,31,112,113], the segregation between PTSD with other medical conditions may limit our understanding of its pathophysiology if observed independently. Further research that investigate the pathogenesis of PTSD through a multi-level approach of inflammatory responses, clinical symptoms, and brain structural and functional changes, may help determine the detailed underlying pathway of PTSD, and open up novel strategies of assessment and intervention for the disorder.

## Figures and Tables

**Table 1:** Studies exploring serum proinflammatory cytokine and C-reactive protein (CRP) level alteration in post-traumatic stress disorder (PTSD).

Trauma Types	Cytokine Markers	N (m/f)	Altered Direction	Main Findings	Ref.
Accident	IL-1β, IL-6, TNF-α	52 (25/27)	Increase	- Individuals with PTSD had higher proinflammatory cytokine concentrations three months after the accident compared to healthy controls, even though clinical symptoms decreased.	[51] ^†^
	IL-1β, IL-6, TNF-α	187 (71/116)	Increase	- Individuals with PTSD had higher IL-1β and TNF-α, and total proinflammatory cytokine scores than those without PTSD.	[25]
				- IL-1β levels in all subjects correlate with self-assessed PTSD symptom severity scores after controlling for trauma exposure severity.	
	CRP	641 (316/325)	Increase	- CRP was positively associated with PTSD severity, particularly with re-experiencing and avoidance symptoms.	[55]
Assault	IL-6	77 (0/77)	Increase	- Individuals with PTSD had higher IL-6 concentrations than trauma-unexposed controls.	[52] ^†^
				- Individuals who recover from PTSD had similar IL-6 concentrations as trauma-unexposed controls.	
	IL-6	68 (0/68)	Increase	- Physical assault history is significantly negatively correlated with phytohemagglutinin A-stimulated IL-6 production.	[41]
	IL-6	60 (10/50)	None	- Individuals with PTSD had statistically similar IL-6 cytokine levels as resilient trauma-exposed individuals.	[56]
Combat	CRP, IL-6, TNF-α	111 (111/0)	Increase	- Individuals with PTSD had lower nitric oxide synthetic capacity and higher CRP levels.	[42]
				- Lower nitric oxide synthetic capacity is associated with higher IL-6, TNF-α, and PTSD symptom severity.	
	IL-6, TNF-α	166 (166/0)	Increase	- Individuals with PTSD had higher levels of IL-6, TNF-α, and other cardiovascular risk markers.	[43]
	IL-6, TNF-α	299 (299/0)	Decrease, Increase	- PTSD symptom severity is correlated with significant decrease in IL-6, increase in TNF-α.	[61]
				- Resilience to PTSD is correlated with increased IL-6.	
	TNF-α	167 (87/80)	Increase	- PTSD symptom severity is positively associated with higher TNF-α levels, and is mediated by attenuated vagal activity, smoking, and alcohol dependence.	[48]
	IL-6, TNF-α	83 (82/1)	Increase	- Individuals with PTSD and TBI had greater increased IL-6 and TNF-α concentrations than individuals with PTSD but without TBI.	[44]
				- IL-6 and TNF-α concentrations were greater in individuals with PTSD and TBI with high PTSD symptom severity than low PTSD symptom severity.	
	IL-1β, IL-6, TNF-α	52 (51/1)	None	- CRP and hair cortisol are correlated with symptoms of depression and PTSD.	[57]
	IL-6	66 (64/2)	None	- Individuals with PTSD and recovered insomnia since deployment had decreased CRP concentration than individuals with PTSD and consistent insomnia.	[58]
	IL-6	143 (138/5)	Increase	- Individuals with PTSD and TBI with loss of consciousness had elevated IL-6 compared to individuals with PTSD and TBI without loss of consciousness as well as individuals with PTSD without TBI.	[45]
	IL-1β, IL-6, TNF-α, IFN-γ	104 (104/0)	Increase	- Individuals with PTSD had higher proinflammatory cytokine levels than those without PTSD.	[15]
				- Proinflammatory cytokine levels were not correlated with symptom severity of depression, PTSD diagnosis, or number of traumas.	
	IL-6, TNF-α	61 (61/0)	Increase	- Individuals with PTSD had higher total proinflammatory scores than those without PTSD after controlling for age, BMI, and smoking.	[16]
				- Total proinflammatory score is not correlated with PTSD symptom severity within the PTSD group.	
	IL-6, TNF-α	13 (12/1)	Increase	- Individuals with PTSD had higher IL-6, TNF-α than trauma-exposed individuals without PTSD.	[46]
	IFN-γ	13 (13/0)	Increase	- Individuals with PTSD had higher levels of IL-6, IL-10, IFN-γ, and TNF-α than healthy controls.	[47]
	IFN-γ	299 (299/0)	Decrease	- Individuals with PTSD showed small but significant decrease in levels of IL-6 and IFN-γ.	[59]
	IFN-γ	30 (27/3) ^‡^	Increase	- Individuals with PTSD had significantly higher IFN-γ levels compared to healthy controls.	[49]
Police	CRP, IL-6	111 (68/43)	None	- There were no significant association between plasma inflammatory markers including levels of CRP and PTSD symptoms.	[60]
Other	IL-6, TNF-α	44 (22/22)	Increase	- Individuals with PTSD had higher IL-6 and TNF-α levels at sleep onset but not at the end of the sleep cycle.	[53] ^†^
				- Men with PTSD show altered levels of TNF-α at the end of the sleep cycle than men without PTSD.	
	IL-6	82 (16/66)	Increase	- Individuals with PTSD had higher IL-6 levels as compared to healthy controls.	[54] ^†^
				- Significant sex-differences were present in IL-6 levels compared to healthy individuals, where men showed higher IL-6 levels than the control group, while women did not statistically differ according to PTSD.	
	CRP	2692 (800/1892)	Increase	- PTSD symptoms were positively associated with high CRP levels.	[50]

^†^ Indicate studies that included a trauma-unexposed healthy control group. ^‡^ Depicts the sample size of the PTSD group only, as gender distribution for the control group was not provided. Abbreviations: BMI, body mass index; CRP, c-reactive protein; f, female; IFN, interferon; IL, interleukin; m, male; N, sample size; PTSD, post-traumatic stress disorder; Ref., reference; TBI, traumatic brain injury; TNF, tumor necrosis factor.

**Table 2:** Studies exploring serum anti-inflammatory cytokines IL-4 and IL-10 alteration in PTSD.

Trauma Types	N (m/f)	Altered Direction	Main Findings	Ref.
Accident	19 (2/17)	None	- PTSD symptom severity is not significantly associated with IL-10 levels.	[68]
	187 (71/116)	None	- There are no differences in levels of IL-10 between individuals with PTSD and those without PTSD.	[25]
Assault	60 (10/50)	Decrease	- Individuals with PTSD presented lower IL-10 levels than the trauma-exposed individuals without PTSD.	[56]
	30 (10/20)	None	- There are no differences in levels of IL-10 between individuals with PTSD and those without PTSD.	[69] ^†,‡^
Combat	167 (87/80)	Increase	- PTSD symptom severity is positively associated with higher IL-10 levels, and is mediated by attenuated vagal activity, smoking, and alcohol dependence.	[48]
	83 (82/1)	None	- There were no anti-inflammatory cytokine level alterations between individuals with PTSD and TBI versus those with PTSD without TBI.	[44]
	64 (60/4)	Increase	- PTSD individuals with mTBI had elevated IL-10 levels compared to individuals with PTSD but without mTBI. - IL-10 concentration is positively correlated with PTSD symptom severity.	[52]
	299 (299/0)	Decrease	- PTSD symptom severity had a trend-line negative correlation with IL-10 levels.	[61]
	52 (51/1)	None	- CRP and hair cortisol are correlated with symptoms of depression and PTSD.	[57]
	143 (138/5)	None	- There are no between-group differences in IL-10 levels among individuals with PTSD and TBI with loss of consciousness versus individuals with PTSD and TBI without loss of consciousness, as well as individuals with PTSD but no TBI. - PTSD symptom severity is not significantly associated with IL-10 levels.	[45]
	61 (61/0)	None	- There are no significant differences in IL-10 levels between individuals with PTSD and those without PTSD.	[16]
	104 (104/0)	None	- Concentrations of IL-10 are not significantly altered in PTSD subjects.	[15]
	13 (12/1)	Decrease	- Plasma and salivary levels of IL-10 are lower in veterans with PTSD compared to veterans without PTSD.	[46]
Refugee	60 (27/33)	None	- IL-10 levels are not significantly different between individuals with PTSD and healthy controls.	[71] ^†^
Other	273 (141/132)	None	- Anti-inflammatory cytokine levels are not different in the chronic PTSD group compared with those in the recovery and resilient group.	[70]
	104 (64/40)	Decrease	- Individuals with PTSD showed increased global DNA methylation and decreased IL-4 than to healthy controls.	[73]
	100 (47/53)	Increase	- Individuals with PTSD showed increased cytokine levels compared to healthy controls in 6 cytokines including IL-2, IL-4, IL-6, IL-8, IL-10, and TNF-α.	[72]
	30 (27/3) ^§^	None	- There are no significant difference in IL-4 levels between individuals with PTSD and healthy controls.	[49]
	82 (16/66)	Increase	- Individuals with PTSD had a significant increase in the serum levels of IL-6 and IL-10 than the control group.	[54] ^†^

^†^ Indicate studies that included a trauma-unexposed healthy control group. ^‡^ Trauma types of the individuals in the PTSD group of this study were described as *other* for two individuals, *car accident* for five individuals, and miscellaneous forms of assault for the remaining individuals. ^§^ Depicts the sample size of the PTSD group only, as gender distribution for the control group was not provided. Abbreviations: CRP, c-reactive protein; f, female; IFN, interferon; IL, interleukin; m, male; mTBI, mild traumatic brain injury; N, sample size; PTSD, post-traumatic stress disorder; Ref., reference; TBI, traumatic brain injury; TNF, tumor necrosis factor.

**Table 3:** Studies exploring PTSD- and oxidative stress-related genetic markers in PTSD.

Trauma Types	Genetic Marker	N (m/f)	Altered Direction	Main Findings	Ref.
Combat	*BDNF*	461 (413/48)	Increase	- Individuals with PTSD had higher frequencies of Met/Met and 66Met allele than individuals without PTSD.	[75]
	Val66Met				
	(rs6265)				
	*BDNF*	257 (257/0)	None	- Individuals had similar frequencies of Met/Met and 66Met allele as resilience trauma-exposed individuals.	[76]
	Val66Met				
	(rs6265)				
Other	*RORA*	435 (260/175)	N/A	- A significant association was reported between a *RORA* SNP (rs8042149) and PTSD diagnosis.	[77]
	SNPs				
	*RORA*	551 (193/358)	N/A	- A significant association was reported between a *RORA* SNP (rs8042149) and PTSD diagnosis.	[78]
	SNPs				

Abbreviations: BDNF, brain-derived neurotrophic factor; m, male; N, sample size; N/A, not applicable; PTSD, post-traumatic stress disorder; Ref., reference; SNP, single nucleotide polymorphism.

**Table 4:** Neuroimaging-based studies exploring oxidative stress and inflammation in PTSD.

Trauma Types	Inflammatory and Oxidative Stress Marker	Outcome Measure (Modality)	N (m/f)	Main Findings	Ref.
Combat	*ALOX12* SNPs, *ALOX15* SNPs	Cortical thickness(sMRI)	218(194/24)	- The *ALOX12* locus (rs1042357, rs10852889) moderated the association between PTSD and reduced cortical thickness in the middle frontal gyrus, superior frontal gyrus, rostral anterior cortex and medial orbitofrontal cortex.	[74]
				- There were no associations between *ALOX15* locus and cortical thickness in PTSD.	
	IL-6, sTNF-RII	Hippocampal volume (sMRI)	206 (170/36)	- Increased sTNF-RII is associated with reduced hippocampal volume.	[87]
				- There were no associations between levels of IL-6 and hippocampal volume.	
				- Higher PTSD severity is associated with elevated sTNF-RII and reduced IL-6 levels.	
Unspecified	IL-1β, IL-6, TNF-α,FDG signal in spleen and bone marrow	FDG signal in amygdala (FDG-PET/CT)	16(10/6)	- There were no associations between IL-1β, IL-6 and TNF-α levels and FDG signal in the amygdala, spleen and bone marrow.	[92]
				- Positive correlations among FDG signals in the amygdala, spleen and bone marrow.	

Abbreviations: CT, computed tomography; f, female; FDG, 18F-fluorodeoxyglucose; IL, interleukin; m, male; N, sample size; PET, positron emission tomography; PTSD, post-traumatic stress disorder; Ref, reference; sMRI, structural magnetic resonance imaging; SNP, single nucleotide polymosphism; sTNF-RII, soluble receptor II for tumor necrosis factor; TNF, tumor necrosis factor.
